# Strong primary care services, an important feature of primary health care: What can Nigeria learn from Israel?

**DOI:** 10.3389/fpubh.2022.1006095

**Published:** 2022-12-16

**Authors:** Akinsola Idowu Akinwumi, Akintayo David OlaOlorun, Stephen Adesope Adesina, Adewumi Ojeniyi Durodola, Isaac Olusayo Amole, Shepherd Roee Singer, Hagai Levine

**Affiliations:** ^1^Department of Family Medicine, Afe Babalola University, Ado Ekiti, Nigeria; ^2^Department of Family Medicine, ABUAD Multi-System Hospital, Ado Ekiti, Ekiti, Nigeria; ^3^Department of Family Medicine, Bowen University, Iwo, Osun, Nigeria; ^4^Department of Family Medicine, Bowen University Teaching Hospital, Ogbomoso, Oyo, Nigeria; ^5^Hadassah Braun School of Public Health, Faculty of Medicine, Hebrew University of Jerusalem, Jerusalem, Israel; ^6^Division of Epidemiology, Ministry of Health, Jerusalem, Israel

**Keywords:** primary care services, primary health care (MeSH), Nigeria, lessons, Israel

## Introduction

The World Health Organization (WHO) described primary health care (PHC) as an “essential health care based on scientifically sound and socially acceptable methods and technology, made universally accessible to all individuals and families in the community through their full participation and at a cost that the community and the country can afford to maintain at every stage of their development, in the spirit of self-reliance and self-determination” (1). According to the Institute of Medicine (IOM) Committee on the Future of Primary Care, 1996, (2) primary care (PC) is defined as the “provision of integrated, accessible health care services by clinicians who are accountable for addressing a large majority of personal health care needs, developing a sustained partnership with patients, and practicing in the context of the family and the community” (2). Its first contact characteristic, and being accessible, comprehensive, integrated, and well-coordinated care delivered with sustained partnership with the individual and/or community served make PC unique (2).

The late Barbara Starfield in her article titled “primary care and equity in health: the importance to effectiveness and equity of responsiveness to peoples' needs” lent credence to both the 2008 World Health Report on PHC by WHO, and the 2007 report of the Pan American Health Organization which described “PHC as those aspects of health policy that make it possible to provide PC services to populations” (3). The foregoing underscores the nature of the relationship between provision of PC services and PHC. Evidence portrayed PC-oriented countries achieve better health indicators (4), and strong PC services help to improve efficiency of the health systems (5).

Nigeria, the most populous African country (6, 7), situated in the West Africa sub-region with an estimated population of over 200 million people (8), has one of the most unfavorable set of health indices in Africa (9), and by extension in the whole world; it constitutes about 1% of the world population, but it is responsible for nearly 13% of the global maternal and under-five mortalities (10). This is partly a consequence of the country's weak PC services and PHC system (11–13). The State of Israel, on the other hand, is a relatively small country with a population of less than 10 million people (14). It is located in the Middle-East at the eastern end of the Mediterranean Sea (15). The country has progressively improved better health indicators (15), and it is reputed for its world-class PC services hinged on strong PC infrastructure (15), and a team of dedicated medical professionals.

Although, the population of Nigeria is more than 20 times that of the State of Israel, the current Gross Domestic Product (GDP) of the former is reportedly smaller than that of the latter, while the GDP of Nigeria as at the year 2021 stood at 440 billion United States Dollars (USD) (16), that of Israel was 481 billion USD (17), which translated into GDP per capita of 2,085 USD (16), and 51,430 USD, respectively (17).

The Israeli health system is supervised by the nation's Ministry of Health and it comprises of an excellent public health effort; a high-level PC services provided by the four health plans (18); specialized ambulatory care provided within the community (14); a topnotch hospital care; and a responsive and effective emergency care (18). The four competing, non-profit-making health plans are saddled with the responsibility of providing a broad package of benefits for every citizen and the permanent residents of the country as stipulated by the government (14). Every citizen or permanent resident of Israel is free to choose from any of the four health plans and has unrestricted access to Primary Care Physicians (PCPs) who help to coordinate referrals to higher level of care whenever the need arises (14).

The major health plan, Clalit has a market share of 53%, Maccabi has a market share of 24%, while the remaining market share of 13 and 10% is contributed respectively by Meuhedet and Luemit health plans (18). About half of the country's acute care hospital beds is owned and operated by the Ministry of Health, the biggest of the four health plans controls another third of the beds, and the remaining beds are managed by a combination of for-profit institutions and not-for-profit organizations (14). In addition, the Ministry of Health also handles the majority of the country's mother and child preventive health centers, about two-thirds of the psychiatric hospital beds and close to 10% of the chronic disease beds (18). Access to care, quality of care, financial stability and equity are ensured by the government with the aid of strict regulations which are directed at the four health plans, hospitals, private insurers, manufacturers and health professionals (14).

Unlike the Israeli health system which is unitarily and centrally supervised by the country's Ministry of Health, probably made possible by the relatively small population and geographical area covered by the country, the Nigerian health system has multiple supervisors such as the Federal Ministry of Health, the State Ministry of Health of each of the 36 states of the Federation including the Federal Capital Territory and the respective Health Department in each of the 774 Local Government Areas recognized by the country's constitution.

In the Nigerian health system, the health care facilities are broadly categorized into three. They are namely; the PHC facilities, the secondary health care facilities, and the tertiary health care facilities. About two-thirds of the health facilities are owned and operated by the government, while the remainder is privately owned, about 88% of these health facilities are classified as primary health facilities, and the secondary and tertiary health facilities constitute about 12 and 0.25% of the health facilities respectively (19). By virtue of the country's health system structure, the provision of PC services principally rests on the over 30,000 PHC facilities located within the country (20, 21). Furthermore, PC was designed to be the closest to the people among the three formally recognized levels of care (primary, secondary and tertiary) and should customarily be the first contact of the citizens with the health care system (11), as it is the case in many of the high- and middle-income countries of the world (2).

In spite of the first contact attribute of PC, it has been documented that about 60–90% of patients bypass the PHC facilities, self-referring themselves to other levels of care with resultant underutilization of PC services and overburdening of the secondary and tertiary health facilities with cases that ought to have been attended to at the PHC facilities (11). As a result, many of the secondary and tertiary health care facilities are sometimes unable to deliver on their mandate of managing advanced medical conditions (due to saturation of beds and occupation of the staff with cases like malaria and diarrhea that should have been managed at the PHC facilities), with eventual undermining of the health care system (11).

According to the WHO's ranking of the world's health systems, the Israeli health system was ranked 28th whereas, the Nigerian health system was placed at the 187th position among the health systems of the 190 countries that were evaluated (22). In the publications derived from the United Nations and the World Bank data, the life expectancy at birth in Israel was 83 years (17, 23), however, the life expectancy at birth in Nigeria is currently 55 years (16, 24). In addition, the infant mortality rate of 2 deaths per 1,000 live births documented for Israel against the year 2022 (25), is significantly lower than the value of 56 deaths per 1,000 live births reported for Nigeria (26). Although, the trend of the life expectancy, infant mortality rate and some other health indices for both countries are apparently in the right direction, the progress recorded by Nigeria is seemingly slow (27).

This narrative review was embarked upon purposely to identify the published literature on the challenges and barriers that need to be surmounted to strengthen the PC services of Nigeria and by extension, the other LMICs in similar situation, through the comparison of the features of the PC services obtainable in Nigeria and Israel with the enthusiasm that the findings thereof will serve as motivation to the relevant health authorities to urgently address the issue of health inequity and help improve the health outcomes of the populations of these countries.

## Methodology

The narrative research technique was employed in this study. Narrative has been described as a critical and interpretative viewpoint that makes use of storytelling (28). A narrative research helps to explore experiences beyond the limitations of questionnaires; offers great insight into how health care services are developed and delivered; and it has been adjudged to be an effective research methodology within and outside the health care industry (29). Sometimes and as it was the case in our study, it helps in the synthesis of knowledge from interrogation of the literatures which might culminate in the better understanding of an identified problem or situation. Probably, the bias of the author(s) of a narrative research could influence its outcomes, however, the fact that this study was executed by multiple authors from different disciplines and separate countries could have helped to reduce such potential bias in this particular work.

We conducted a literature search of the electronic databases of the World Health Organization, Directory of Open Access Journals, Digital Commons Network, Pubmed, Google Scholar, Public Library of Science, BioMed Central, Web of Science, and ScienceDirect for the articles published mainly on the health services of Israel and Nigeria over the last two decades (between 2003 and 2022). The key search terms used included “primary health care,” “primary care,” and “World Health Organization building blocks” (the search of these was not limited to Israel and Nigeria). The additional key words whose search were restricted to Israel and Nigeria were “health care policy and legislation,” “health workforce,” “health management information system,” “access to quality medicines,” “health care financing,” “health indicators” and “Gross Domestic Product.”

The authors utilized the Health System Building Blocks Framework (30), to make a comparison between the health systems of the two sovereign states of Israel and Nigeria. This Framework for Action tagged “Everybody's business: Strengthening Health Systems to Improve Health Outcomes” was developed by the WHO in the year 2007 primarily to urgently improve the performance of health systems by helping to “…promote the common understanding of what a health system is and what constitutes health systems strengthening” (31). Since its introduction as a means of assessing the performance of the health care systems, it has been reported that the WHO Health System Building Blocks Framework is capable of tackling the existing problems militating against the strengthening of the overall health system, if properly harnessed; and it could also serve as an impetus for actualizing the global health targets like the Sustainable Development Goals (30).

The Framework has six components that are used for the evaluation of the health systems, and these include service delivery, health workforce, health information management system, access to essential medicines, health care financing and leadership and governance (30). After comparing the PC services obtainable in both countries, important lessons for Nigeria and other low- and middle-income countries (LMICs) were drawn and policy implications were subsequently highlighted.

## The six components of the WHO Health System Building Blocks Framework

The six components of the WHO Health System Building Blocks Framework that were used to compare the PC services between the two countries are discussed below;

### Service delivery

There is wide geographical spread of clinics and health centers rendering PC services across the State of Israel (32). The same applies to Nigeria (13), which has over 30,000 PHC facilities (20, 21), extensively located in different parts of the 9,565 political wards of the 774 local government areas of the country (21). The four Israeli health maintenance organizations (HMOs) supply PC services to the inhabitants of Israel under the supervision of the country's Ministry of Health (32). They also include home care services for temporarily and permanently homebound patients (33). In Nigeria, provision of PC services is largely by the government PHC facilities supported by private facilities widely spread across the country. While some of the private facilities provide home care services, the government health centers that constitute the majority of the PC service providers, hardly render home care services, thereby making seamless transition of patients to their homes following their discharge from the hospital a herculean task. Consequently, the fate of homebound patients significantly rests on their relatives.

The provision of PC services at the clinic and health centers in Israel (32), helps to clearly demarcate the delivery of PC services from urgent, emergency and hospital care; and enables both the Non-Physician Primary Care Providers (NPPCPs) and PCPs to focus on the delivery of quality PC services. Conversely, in Nigeria, urgent, emergency and hospital (inpatient) care are also provided by many of the PHC facilities. This extra burden is capable of hampering their effectiveness in delivering PC services to the population. Although, the secondary and tertiary health care facilities are purposefully designed, built, and equipped with the intention of handling the health care problems that the PHC facilities are unable to address, to a very limited extent, the General Outpatient Departments located within the secondary and tertiary health care facilities also render PC services.

The quality of the PC services received by Israeli citizens is reportedly high and many of the consumers are satisfied with their experience of the PC system (33). The reverse is the case in Nigeria, where citizens generally lack confidence in the quality of services provided at the PHC facilities (12), hence, the high occurrence of cases of self-referral to other levels of care. In Israel, the PC providers are equally highly motivated to render health promotion and disease prevention as important aspects of PC services, while this is mostly lacking in Nigeria (13). For the greater number of the patients in Israel, the waiting time before being attended by their PC provider is below 15 mins (33), in Nigeria, the PHC facilities are characterized by long waiting hours, with almost all the patients spending more than an hour in the health facilities prior to seeing a doctor (34).

Since the enactment of the Israeli national health law (NHL) in 1995, all inhabitants of Israel are eligible recipient of health care services provided by the country's four HMOs in any of the PC clinics located in the country (32). The compulsory basic health insurance for all citizens (35), and minimal use of fee-for-service mode of payment for PC services are responsible for good financial access to PC services in Israel (33). Contrarily, in Nigeria, >90% of population pays for health care using the Out-of-Pocket (OOP) modality (36), while the National Health Insurance Scheme (NHIS) covers < 5% of the population (36), because as it stands now, most of the people enrolled in the scheme are the civil servants and some of their dependents. Although, the federal, state and local governments often claim PC services are free, this is not the reality in practice, where citizens have to pay often exorbitant fees before they could receive PC services mainly because of perennial scarcity of drugs and other consumables (13). This remarkably constricts access to PC services delivered in Nigeria.

### Health workforce

The PC workforce in Israel comprises of nurses, other non-physician health care workers and PCPs (such as family medicine, pediatrics and internal medicine physicians) that are widespread all over the country (33). In Nigeria, the PC practitioners include community health extension workers (represents the bulk of the health workers in Nigerian PC), nurses, other non-physician health workers, and PCPs (mainly community health physicians and a few family physicians) with high concentration of PCPs at the urban centers. The Israeli PC is relatively better staffed; and the PC personnel discharge their duties under a more conducive atmosphere with high level of professionalism (33). Furthermore, unrestricted access to PCPs and a well-developed PC system where a certain number of citizens are allocated to a PCP for the purpose of providing quality PC for them are some of the other factors that have been adjudged responsible for the efficiency in the Israel's health system (14). These enable the PCPs to know their patients and their families inside out and also give them the advantage of detecting and resolving problems promptly.

Some of the staffing problems confronting PC services in Nigeria include shortage of qualified medical personnel (12), adverse working conditions, misdistribution of health care workers, brain drain, and inter-professional rivalry amongst others (7). For instance, according to the World Bank data, the Nigerian physician-people ratio and nurse/midwives-people ratio are 0.4–1,000 (37); and 1.5–1,000 respectively in 2019 (38). These statistics are much better in Israel which boasts of physician-people ratio of 5.5–1,000 (37); and nurse/midwives-people ratio of 6.6–1,000 respectively in 2019 (38). Furthermore, compared with their community health counterparts, family physicians are yet to be fully integrated into PC (which is supposedly their domain) in Nigeria, the pediatricians and internists are completely absent at the PHC facilities in the country.

### Health management information system

Another important feature of the Israeli health system that probably makes it to be able to stand out is the digitalization of the entire medical records. This helps to improve efficiency and effectiveness of the health system since the medical record of any patient could be accessed by the authorized medical professional when necessary (14). The computerization of the patient medical records in Israel led to improved access to the up-to-date patients' medical history, investigation results, the previous consultations and hospitalizations (32). This also enhances information sharing nationwide, quality of care and reduction of cost of care (33), and helps in the development of many quality indicators for improvement of the national health system (33).

In Nigeria, the challenge of low usage of information communication technology (ICT) such as electronic medical record (EMR) system persists (39), and almost all, if not all the PHC facilities still rely on the use of paper and pen to carry out their daily activities. This makes record-keeping, access to patients' information and sharing of information between hospitals and professional colleagues extremely difficult.

### Access to essential medicine

The importance of prompt access to safe, effective, quality and affordable essential medicines (EMs) by all the people that need them cannot be overemphasized (40). Israel had encountered problem of access to EM in the past (41). The factors that were responsible for the problem of drug shortage were identified by the appropriate stakeholders and measures that addressed them such as guidelines ensuring the Ministry of Health is promptly notified of any imminent shortage of drugs by the Marketing Authorization Holder (MAH), stoppage of further lowering of drug prices below 17 new Israeli shekels, and ordering all MAHs to maintain at least 1 month stock of all registered and non-registered drugs in Israel amongst others were put in place (41). Moreover, the compulsory, non-discriminatory, non-profit health insurance system in Israel enables every Israeli citizen and the permanent residents to have unrestricted access to the basic health package/plan that provides prescription drugs and other available services (35).

In Nigeria, EMs are not readily available at the facility level (13). Some of the barriers to availability of EMs in Nigeria include poor financial commitment of government at all levels to adequate funding of PHC (13), epileptic supply of essential drugs to the facilities (12, 13, 42), high cost of authentic drugs (43), infiltration of the Nigerian drug market by adulterated medicines, and poor pharmaceutical regulatory mechanisms (44), with attendant hampering of smooth delivery of quality PC services (13). The situation is so alarming such that patients could procure prescription drugs from drug retail outlets without prescription by a qualified medical personnel due to poor regulatory oversight function of relevant authorities with resultant wastage of scarce resources, getting inappropriate treatment, delay in making the right diagnosis and occurrence of needless complications (44).

### Health care financing

Israel has built an equitable health system at relatively minimal health spending of about 7.5% of the GDP (45), which is below the Organization for Economic Cooperation and Development (OECD) average of 8.9% (45, 46). Despite the fact that the country spends below 8% of its GDP on health care (14), the Israeli health care system is efficient (14), and Israel enjoys health indices that are at par with the health parameters of the other developed nations whose annual health expenditures are above that of Israel (14, 15, 46). This relatively low health spending was realized by keeping the OOP method of payment for health care at its barest minimum with the introduction of the compulsory progressively financed statutory health insurance system in 1995, which enabled Israel to run an all-inclusive health care that leaves no Israeli behind (14).

This enjoyment of universal access to PC services by the citizenry occurs irrespective of the citizens' educational background, social status, financial capability, ethnicity, religious or political affiliation (15). The Israeli PC financing is further characterized by insignificant usage of fee-for-service mode of payment by all the four HMOs and complete absence or minimal use of co-payments for PC services in majority of the HMOs which rarely constitute barrier to access health care (33).

In 2019, Nigeria's health care spending was relatively low, with total health expenditure of about 71 United States dollars (USD) per capita (47), which was only about 3.03% of the GDP (48), whereas, during the same year, Israel spent 3,456 USD per capita (47), which stood at about 7.46% of GDP on health (48). The two major sources of health care financing in Nigeria are tax-based revenues and private contributions (from organizations and individuals) (49). A part of the revenue accrued to the federal government is allocated to the three tiers of government (federal, state and local) monthly, while the federal government's allocation is paid into its account, the share of both the state and local governments is paid into a joint account under the control of the respective state governor, and the allocation is later shared between the state and the various local governments under it (13). However, lack of transparency and accountability as well as administrative and political bottlenecks at the state government level often result in underfunding of the PC services which is traditionally under the local government control, as the chunk of the meager available funds eventually received by the local governments is committed to payment of salaries, with little or nothing left for procurement of drugs and consumables and other operational costs (13).

Additionally, OOP payment remains the main method of payment for health care (13, 36), as more than 90% of Nigerians pay for health care OOP (36). The NHIS was launched in 2005 to address OOP mode of payment challenge in a bid to prevent and/or mitigate catastrophic health expenditure among the citizenry. Surprisingly, the Nigerian NHIS that started in 2005, only about 10 years after the Israeli NHL came into force, is yet to cover more than 5% of the population (50). This contributes to poor financing of PC services in Nigeria, because a significant number of the citizens live below poverty line (51).

### Leadership and governance

The existence of a health care system that is accountable to the people in the State of Israel makes all the difference. This has propelled various relevant stakeholders in delivery of PC services under the headship of the Ministry of health to continually work tirelessly to guarantee the Israel population of a state-of-the-art PC services by strengthening the institutions involved in the delivery of PC services in the country through ceaseless development of PC services quality monitoring indicators for more than two decades (52), with resultant improvement of the national health system (32). The country is also reputed for its excellent medical academic culture, well equipped hospitals, strong PC infrastructure, and yearly upgrade of the national basket of health services (15).

The deployment of the four health plans that are well regulated by the government to render PC services to the population with broader system perspective and information technology has further improved organization of PC services in the country (33). The ability of the authorities to ensure pay parity between PCPs working at the peripheral and municipal centers and keep the health care spending relatively low are also some of the important factors that account for strong PC services and better health indices in the country (33).

In Nigeria, there are many governance and leadership factors militating against smooth delivery of PC services in the country. Some of these include high cost of services, inadequate health infrastructure (12), and remuneration disparity between different levels of care, inadequate training facilities (7), poor community involvement (12), absence of accountability and transparency in the day-to-day running of the health system, duplication of efforts by many governmental and non-governmental organizations, entrenchment of vertical programs, and low political commitment to implementation of approved health policies (7).

## Lessons for Nigeria and other low- and middle-income countries

The Table 1 contains a summary of the lessons from the Israeli PC services that could be emulated by Nigeria and other LMICs for the benefit of their health systems and teeming populations.

**Table 1:** Summary of the lessons from Israeli primary care services.

Unrestricted access to NPPCPs and PCPs in a well-developed PHC system helped to improve the use of PC services by the citizens
Digitalization of the medical records led to improvement in efficiency and effectiveness of the PC system and also helped to lower cost of care
Investment in PC personnel, and infrastructure resulted in a strong PC services
Annual upgrade of the national basket of health services covered by health insurance led to expansion of PC services
Enactment of statutory health insurance system that is progressively financed increased access to PC services
Availability of funding for excellent medical and academic research strengthened PC service delivery
Continuous development of community quality indicators for monitoring health system performance improved the quality of PC services in Israel
Unwavering commitment to PC services serves as a veritable means of achieving UHC

*NPPCPs, Non-Physician Primary Care Providers.

*PCPs, Primary Care Physicians.

*PHC, Primary Health Care.

*PC, Primary Care.

*UHC, Universal Health Coverage.

### Policy implications for Nigeria

For Nigeria to appropriately address and reverse many, and perhaps all of the unfavorable health indicators, the commitment of the government to timely institutionalization of regulations capable of stirring-up a paradigm shift in the delivery of PC services by promoting the core values of PC and PHC in the country is non-negotiable.

Efforts of the government and various stakeholders in PC should be geared toward ensuring suitable policies and legislations are put forward and judiciously implemented to ascertain care is properly coordinated from the tertiary and secondary health care facilities back to the PHC facilities as well as from the specialists back to the PC providers when indicated. This is aside the enforcement of the gatekeeping role of the PCPs in the initiation of referral from PC to other levels of care.

This bi-directional feature of the referral system has strong tendency of facilitating decongestion of the tertiary and secondary health care facilities and enable them focus on the main goal for their establishment and also allow the specialists to concentrate on the essence of their training. To further ensure proper coordination of care, mandating every patient (with exceptions of dire emergencies) to pass through the PHC facility as the first contact with the health system and thereafter allow the PCPs to determine the merit of their referral to other levels of care is necessary. In addition, clear delineation between institutions that provide PC services and those rendering urgent, emergency and hospital care is also advocated.

Against the current backdrop, where PCPs are largely employed at the secondary and tertiary health facilities (both private and public), we support policy that encourages the deployment of the PCPs mainly at the PHC facilities to augment the workforce in these facilities and further strengthen the coordination of PC services in the country for greater efficiency and better service delivery.

In addition, allocation of certain number of patients to the care of a PCP is also recommended, as this has the potential of addressing the problem of doctor-shopping in Nigeria. We as well, advocate proper recognition and integration of home care services into health care system in Nigeria to ensure continuity of care. Computerization and centralization of payment of health care workers to improve transparency and accountability; and financial motivation of those practicing in the rural communities in order to increase efficiency and productivity are advised. We equally encourage incentivizing those rendering health promotion and disease prevention as integral parts of PC services.

Adoption of EMR system for PC services to enable easy access to patients' records and data for monitoring, evaluation and research, and medical consultations is recommended and follow-up of patients *via* telemedicine is alike encouraged. Constitution of functional drug revolving committee in every PHC facility to deal with the incessant problem of “out-of-stock syndrome” by ensuring regular supply of essential drugs and efficient management of drug revolving funds is solicited.

The law that established NHIS should be urgently reviewed so as to make social health insurance compulsory for all Nigerians in order to increase financial access to PC services, fast-track universal health coverage, as well as prevent catastrophic health spending (CHS) and impoverishing out-of-pocket spending. Expanding the NHIS services and coverage in Nigeria, encouraging financial probity, instilling culture of stewardship in various stakeholders, ensuring greater commitment of the government to the insurance scheme, involvement of community heads in the day-to-day running of the PHC facilities, sensitization of the populace on the importance of utilization of available PC services, NPPCPs and PCPs are also considered germane for better future of PC service delivery in Nigeria.

## Conclusion

The strenuous impact of the COVID-19 pandemic on the health system of many countries has further stressed the significance of an excellent PHC that is equitably accessible to individuals from various strata of the society (53, 54). It is equally evident that strong PC services help to improve efficiency of health systems, and the overall health indicators (55, 56), especially in a resource-poor setting (55), like Nigeria.

It is crystal clear that there is so much to be learnt from the Israeli health care system and PHC in particular, and more precisely from its tradition of provision of equitably accessible topnotch PC services to its population. However, it is important to state unequivocally that the country does not regard its PC services as perfect and measures are in place to continually improve upon the services. It is advised that Nigeria and other LMICs yearning for a better PHC as a way of laying a solid foundation for an overhauled health care system should apart from taking a cue from Israel and many other nations with functional PC services, consider a whole system perspective that takes into cognizance the differences in their context when domesticating the lessons that could be imbibed from Israel and the other countries of the world with working PC services. The authors are optimistic that the adaptation and/or proper application of some or all of the suggestions enumerated above would help many of the LMICs whose prevailing PC services situation mirrors that of Nigeria to measurably improve their health system significantly in the coming years.

## Author contributions

AIA conceived and wrote the original manuscript, while SRS, ADO, and HL supervised the work. All authors made substantive contributions to the initial and final drafts of the manuscripts, read, approved, and agreed to be responsible for their contributions.
